# Research on a Transformer Vibration Fault Diagnosis Method Based on Time-Shift Multiscale Increment Entropy and CatBoost

**DOI:** 10.3390/e26090721

**Published:** 2024-08-23

**Authors:** Haikun Shang, Tao Huang, Zhiming Wang, Jiawen Li, Shen Zhang

**Affiliations:** Key Laboratory of Modern Power System Simulation and Control & Renewable Energy Technology, Ministry of Education, Northeast Electric Power University, Jilin 132012, China; 949080280@neepu.edu.cn (T.H.); 2202300311@neepu.edu.cn (Z.W.); 2202300208@neepu.edu.cn (J.L.); 2202300135@neepu.edu.cn (S.Z.)

**Keywords:** transformer, vibration signal, time-shift multiscale increment entropy, CatBoost, fault diagnosis

## Abstract

A mechanical vibration fault diagnosis is a key means of ensuring the safe and stable operation of transformers. To achieve an accurate diagnosis of transformer vibration faults, this paper proposes a novel fault diagnosis method based on time-shift multiscale increment entropy (TSMIE) combined with CatBoost. Firstly, inspired by the concept of a time shift, TSMIE was proposed. TSMIE effectively solves the problem of the information loss caused by the coarse-graining process of traditional multiscale entropy. Secondly, the TSMIE of transformer vibration signals under different operating conditions was extracted as fault features. Finally, the features were sent into the CatBoost model for pattern recognition. Compared with different models, the simulation and experimental results showed that the proposed model had a higher diagnostic accuracy and stability, and this provides a new tool for transformer vibration fault diagnoses.

## 1. Introduction

Transformers, as a key type of equipment in power systems, are responsible for voltage-regulation and electricity-transmission tasks. They play a crucial role in ensuring the stability of the entire power grid [1]. However, the transformer operation needs to be continuous, and the complex environment increases the probability of failure. Once a transformer fails, it may impact both the power system and socioeconomic development [2]. Therefore, to ensure the long-term stability of the power system, it is necessary to promptly detect faults in transformers and monitor their real-time operational status. In recent years, transformer fault detection techniques are becoming increasingly diverse. According to previous statistical reports, the majority of transformer faults are caused by the mechanical degradation of windings and cores [3]. The detection of these mechanical faults can be achieved by analyzing vibration signals on the surface of the transformer enclosure. A vibration analysis does not require a direct electrical connection to the transformer. The signal acquisition equipment is simple and easy to carry, thus avoiding any disruptions to the normal operation of the power system.

Feature extraction is crucial for a transformer fault diagnosis based on a vibration signal analysis, and it directly affects the accuracy of subsequent diagnostic results. Reference [4] converted transformer vibration signals into time–frequency images using a short-time Fourier transform (STFT) and determined the transformer’s operational status based on the time–frequency domain signal characteristics. Reference [5] used the mean, median, and standard deviation of the fundamental and harmonic components’ amplitudes of the transformer vibration signals as health indicators to diagnose mechanical faults in the transformer windings and cores. Reference [6] extracted 19 features from the transformer vibration signals, including the mean, maximum, root mean square, variance, and kurtosis. The Fisher score and a linear discriminant analysis (LDA) were employed to select and reduce the dimensionality of these features. Then, a support vector machine was utilized to accurately identify the various states of the transformer. Reference [7] proposed a feature extraction method by combining a synchrosqueezed wavelet transform (SWT) and a synchrosqueezed generalized S-transform (SSGST). By converting transformer vibration signals into images and using transfer learning algorithms to optimize deep learning networks, the accuracy of transformer fault diagnoses was improved. Reference [8] applied a hierarchical dynamic aggregation graph (HDAG) to convert transformer vibration signals into two-dimensional time–frequency images, extracting the features of different transformer fault signals and achieving a high fault recognition accuracy. The above methods extracted the features of the transformer vibration signals through a spectral analysis, requiring a transformation from a time domain to a frequency domain. However, after obtaining the time- and frequency-domain features, these features may overlap. If the selection criteria are unreasonable, feature duplication or important feature losses may occur. In vibration signals, due to the nonlinear characteristics, the time-domain and frequency-domain graphs of different faults may overlap, making it difficult to distinguish the extracted features [9]. A nonlinear dynamic analysis, as another important feature extraction method, measures the signal complexity using the entropy, thereby extracting the dynamic and complexity features of the signal. Compared to a spectral analysis, this method does not require a time–frequency domain transformation, enabling the better capture of the signal’s nonlinear dynamic features. It provides a more comprehensive description of the signal features and requires no prior knowledge. Therefore, it has been widely applied for feature extraction in various fields. Since Shannon entropy was proposed in 1984 [10], many scholars have proposed various entropy types according to different feature requirements, such as approximate entropy [11], sample entropy [12], fuzzy entropy [13], permutation entropy [14], and dispersion entropy [15], which have been widely applied in signal feature extraction. However, a single entropy value cannot comprehensively extract signal feature information. Therefore, Costa et al. [16] proposed the concept of multiscale entropy. Since then, many scholars have begun research on the design and improvement of multiscale entropy in the field of feature extraction. Reference [17] proposed time-shift multiscale fuzzy entropy and combined it with a Laplacian support vector machine to diagnose the faults in rolling bearings. Reference [18] conducted the online monitoring of railway vehicle suspension systems using multiscale permutation entropy and a linear local tangent space alignment. Reference [19] applied the improved composite multiscale dispersion entropy to monitor electroencephalogram signals, achieving the effective detection of epileptic seizures. The methods proposed in the above literature have achieved good application effects in signal feature extraction, but different entropy types have a diverse performance in describing the signal characteristics in various fields. Transformer vibration signals, as a type of nonlinear and non-stationary signal, exhibit increases or decreases in fundamental and harmonic components under normal and faulty conditions, leading to dynamic changes throughout the entire time series. Therefore, in order to extract the accurate characteristics of transformer vibration signals, it is necessary to select feature indices that can accurately represent the different operating states of the transformer.

Increment entropy, which was proposed by Liu et al. [20], effectively detects changes in the complexity of physiological time series and distinguishes different sequences, serving as a novel measure of time-series complexity. This index applies symbolic dynamics to time series, quantifying the change degrees between adjacent elements of symbolic sequences, thereby better characterizing the structural information in time series and capturing the dynamic characteristics [21]. Reference [22] measured the time series at multiple scales using multiscale increment entropy (MIE). However, traditional coarse-graining methods led to the loss of effective information as the scale increased, resulting in the decreased stability of entropy. For this reason, this paper introduces the notion of a time shift and proposes time-shift multiscale increment entropy (TSMIE). This index integrates the fractal theory with the entropy theory, using the Higuchi fractal dimension to construct a set of new time series with fractal curve properties at the original time scale. This approach not only explores the irregularity of the time series in depth, but also effectively preserves the important structural information of the original data [23]. Moreover, TSMIE inherits the advantages of incremental entropy and accurately reflects the dynamic changes of signals by combining their time–frequency characteristics, thus providing a more stable and accurate description of time-series signal features. Therefore, this paper attempts to employ TSMIE to extract the vibration signal features of different faults in transformers.

Pattern recognition is the next crucial step in a transformer fault diagnosis. In recent years, with the continuous development of artificial intelligence, machine learning algorithms have been widely applied in the field of fault diagnoses due to their excellent reliability, strong generalization ability, and robustness, achieving good application effects. Machine learning methods such as a back-propagation neural network (BPNN) [24], a support vector machine (SVM) [25], an extreme learning machine (ELM) [26], and K-nearest neighbor (KNN) [27] have achieved good diagnostic results in pattern recognition. The advantages and disadvantages of traditional machine learning algorithms are summarized in Table 1.

It can be seen from Table 1 that traditional machine learning algorithms have certain shortcomings, hindering their further development in the field of pattern recognition.

Ensemble learning algorithms, based on the principles of collective intelligence and complementary advantages, construct a strong learner by combining multiple weak ones, effectively improving the model’s generalization ability, accuracy, and robustness. Compared to traditional single classifiers, ensemble learning methods have a better performance and better application effects, making them a hot research topic in the field of machine learning. CatBoost, as a representative ensemble learning algorithm, adopts improved categorical feature-handling algorithms and greedy strategies, which can efficiently handle categorical features. Additionally, the proposed ranking boosting theory effectively addresses the gradient bias and prediction shift issues [28]. It possesses an excellent generalization ability and exhibits a robustness to outlier data and noise. Based on this, this paper attempts to use the CatBoost algorithm to diagnose different fault modes of transformers.

In summary, considering the characteristics of transformer vibration signals, this paper proposes a transformer fault diagnosis model based on the combination of TSMIE and CatBoost. The main innovations of this work are as follows. Firstly, by addressing the issues of cumbersome feature extraction steps and the large number of extracted features, this paper proposes a new indicator for measuring the complexity of temporal signals named TSMIE. The impacts of various parameters on TSMIE were investigated through simulated signal studies, confirming the excellent performance of the proposed index in feature extraction. Secondly, TSMIE was applied to extract features from the vibration signal of the transformer, and the results showed that the obtained features could effectively distinguish between normal and fault states of the transformer. Finally, the obtained features were input into the CatBoost classifier to form the TSMIE–CatBoost diagnostic model. Through a comparative analysis with different fault diagnosis models, the results showed that the model had the best fault diagnosis performance, verifying the superiority of the proposed method.

## 2. TSMIE–CatBoost

### 2.1. Theory Background

#### 2.1.1. Incremental Entropy

Incremental entropy can better characterize the structural information in time series. It captures the dynamic characteristics of signals by applying symbolic dynamics to time series and quantifying the increments between adjacent elements in the symbolic sequence. This indicator maps each increment to a character consisting of two letters that contain the symbol information and amplitude information. It considers the rate and the temporal sequence of signal changes. It is sensitive to the changes in different frequency components of the signal and the capture of temporal information, which can better characterize the structural information in the time series. The basic principle of incremental entropy is as follows.

For the original time-series signal, X={x(i) , i=1,2,⋯,N}. N is the data length.

(1) Firstly, an incremental time series is constructed:

(1) v(i)=x(i+1)−x(i) 

Subsequently, the incremental time series is divided into N−m incremental vectors with dimensions of m:

(2) V(h)=[v(h),⋯,v(h+m−1)] , h=1,2,…,N−m

where m is the embedding dimension, m≤N.

(2) Each element in the incremental vector V(h) is mapped to a character $s_{h+l}q_{h+l}$ consisting of two elements according to the following rules:

(3) $$s_{h+l}=\mathrm{sgn}\left(v(h+l)\right),\;l=1,2,\ldots ,m-1$$

(4) $$q_{h+l}=\mathrm{size}\left(v(h+l)\right)=\{\begin{array}{ll} 0, & std(V)=0\\ \min\left(R,\frac{\|v(h+l)\|\times R}{std(V)}\right), & std(V)\neq 0 \end{array}\;,\;l=1,2,\ldots ,m-1$$

where $s_{h+l}$ is the incremental symbol, describing the fluctuation direction between corresponding adjacent elements in the coarse-grained time series. Values of 1, 0, and −1 indicate that the changes between adjacent elements are either increasing, unchanged, or decreasing. R is the resolution parameter. ‖v(h+l)‖ is the norm of a vector. $q_{h+l}$ is the incremental size, describing the magnitude of changes between adjacent elements, whose value depends on the resolution parameter R. std(V) is the standard deviation of the incremental vector sequence.

(3) According to step (2), V(h) is mapped to a character with 2m elements:

(5) $$w_{h}=U_{l=0}^{m-1}s_{h+l}q_{h+l}$$

For the time series v(i), N−m character sets can be obtained. Given m and R, each character has ${\left(2R+1\right)}^{m}$ variant patterns. Therefore, the relative frequency of each unique character is defined as follows.

(6) $$q\left(w_{g}\right)=\frac{Q\left(w_{g}\right)}{N-m}$$

where $w_{g}$ is each unique character in the character group $w_{h}$, and $Q\left(w_{g}\right)$ is the total number of unique characters in $w_{h}$.

(4) Therefore, the incremental entropy on a single scale is defined as follows:

(7) $$\mathrm{IE}(X,m,R)=-\sum_{i=1}^{{\left(2R+1\right)}^{m}}q\left(w_{g}\right)\log q\left(w_{g}\right)$$

#### 2.1.2. Multiscale Increment Entropy 

In order to comprehensively reveal the dynamic characteristics of signals and improve the accuracy and robustness of signal processing, the time series can be coarse-grained to obtain the multiscale increment entropy. The principle of the MIE algorithm is as follows.

For the original time-series signal X={x(i), i=1,2,⋯,N}, coarse-grained processing is performed to obtain a coarse-grained time series.

(8) $$y_{j}^{\tau }=\frac{1}{\tau }\sum_{i=(j-1)\tau +1}^{j\tau }x(i),\;1\leq j\leq \left\lfloor \frac{N}{\tau }\right\rfloor$$

where τ is the scale factor and $\left\lfloor \frac{N}{\tau }\right\rfloor$ is rounding to $\frac{N}{\tau }$.

The scale factor is τ=1,2,3,⋯. When τ=1, the coarse-grained time series is equal to the original time series. When τ>1, the original time series is divided into τ coarse-grained time series of length $\left\lfloor \frac{N}{\tau }\right\rfloor$.

By calculating the incremental entropy of each coarse-grained time series, the multi-scale increment entropy can be obtained.

(9) $$\mathrm{MIE}(X,m,R,\tau )=\mathrm{IE}(y_{j}^{\tau },m,R)$$

#### 2.1.3. Time-Shift Multiscale Increment Entropy

Due to the limitations of multiscale entropy when used with a coarse-grained time series, some effective information may be lost, resulting in entropy bias. Therefore, this article introduces a time-shift operation, combining fractal theory with entropy theory. The Higuchi fractal dimension was employed to construct a new time series with fractal curve properties on the original time scale. This phase distribution operation can not only deeply explore the irregularity of time series, but also effectively preserve the important structural information of the original data. The proposed TSMIE index considers the dynamic changes and temporal characteristics of signals on the time axis. Compared to multiscale increment entropy, this approach can comprehensively describe the time-varying characteristics and local features of signals, enabling a more accurate analysis of the signals at different time scales. The principle of TSMIE is as follows.

**Figure 1:**
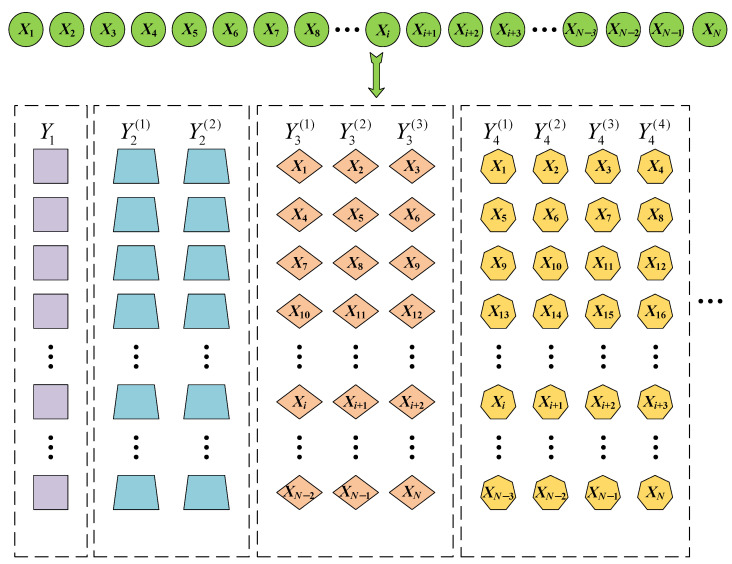
The construction process of time-shifted subsequences.

(1) For the original time-series signal X={x(i) , i=1,2,⋯,N}, time-shifted subsequences are constructed based on the theory of Higuchi fractal dimension.

(10) $$Y_{k}^{\beta }=\{x_{\beta },x_{\beta +k},x_{\beta +2k},\ldots ,x_{\beta +\Delta }\}$$

where k is the time-shift scale factor (i.e., time interval), β is the initial time point, and Δ denotes rounding to the nearest integer for (N−β)/k. To illustrate the construction process clearly, the construction of time-shifted subsequences is shown in Figure 1.

(2) Each time-shifted subsequence is reconstructed as follows.

(11) Z={z(α) , α=1,2,…,Δ}

where $z(1)=x_{\beta },z(2)=x_{\beta +k},\ldots ,z(\alpha )=x_{\beta +\Delta }$.

(3) An incremental time series is constructed for each time-shifted subsequence in each time-shifted sequence set.

(12) $$v^{\prime }(j)=z_{\alpha }(j+1)-z_{\alpha }(j)\;,\;j=1,2,\ldots ,\Delta -1$$

Then, the incremental time series is divided into Δ−m incremental vectors.

(13) $$V^{\prime }(h)=[v(h),\cdots ,v(h+m-1)],\;h=1,2,\ldots ,\Delta -m$$

where m is the embedding dimension.

(4) Each element in the incremental vector $V^{\prime }(h)$ is mapped to a character ${s^{\prime }}_{h+l}{q^{\prime }}_{h+l}$ composed of two elements according to Equations (3) and (4).

(5) According to Equation (5), $V^{\prime }(h)$ is mapped to a pattern vector $w_{h}^{\prime }$ with 2m characters. For the time series $v^{\prime }(j)$, Δ−m character sets are obtained, and for the given m and R, each pattern vector has ${\left(2R+1\right)}^{m}$ variant patterns. Therefore, according to Equation (6), the relative frequency $q^{\prime }\left({w^{\prime }}_{g}\right)$ of each unique character is calculated.

(6) For a given time scale τ, the average incremental entropy of all the time-shifted subsequences within the range 1≤β≤k is the time-shift multiscale increment entropy, shown in Equation (14).

(14) $${\mathrm{TSMIE}}_{1\leq k\leq \tau }(X,m,R,\tau )=\frac{1}{k}\sum_{\beta =1}^{k}\mathrm{IE}(Y_{k}^{\beta },m,R)$$

In summary, the TSMIE pseudocode is shown in Algorithm 1.
**Algorithm 1** Time-shift multiscale increment entropy.**Input:** (1) X: Time series    (2) m: Embedding dimension    (3) R: Resolution parameter    (4) τ: Scale factorRunning process:1 for k=1,2,…,τ do2  Construct time shifted subsequences $Y_{k}^{\beta }$, obtaining the set of time shifted sequences Z
3    for β=1,2,…,k do4     Construct incremental time series in each time shifted sequence set $v^{\prime }(j)$
5     Divide incremental time series into incremental vectors $V^{\prime }(h)$
6     Map $V^{\prime }(h)$ to ${w^{\prime }}_{h}$
7     Calculate the relative frequency of each unique character $q^{\prime }\left({w^{\prime }}_{g}\right)$
8     Calculate the IE9    end for10  Calculate TSMIE11 end for**Output:** TSMIE values

### 2.2. Influence Analysis of TSMIE Parameters

**Figure 2:**
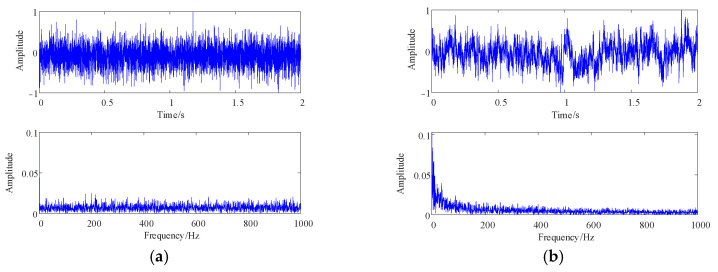
The simulation signal. (**a**) White noise; (**b**) pink noise.

TSMIE is influenced by parameters such as the data length N, the embedding dimension m, the resolution parameter R, and the time scale τ. Different parameter settings can affect the accuracy of subsequent feature extraction. Therefore, in this section, the simulated signals were used to analyze the effects of different parameters on TSMIE. Transformer vibration signals under different faults may have different frequency components, which will cause differences in their time-domain plots. Gaussian white noise and pink noise in the simulated signals had image characteristics similar to transformer vibration signals due to their different energy distribution spectra. Therefore, this paper selected Gaussian white noise and pink noise for analyzing the influence of the parameters on TSMIE. The time and frequency domain diagrams of the simulated signals are shown in Figure 2.

From Figure 2a, it can be observed that white noise exhibited random fluctuations without obvious periodic structures. The amplitude between each sampling point was random, showing no clear trend. Moreover, white noise had a uniform energy distribution across the entire frequency spectrum, with relatively equal energy at each frequency and no apparent frequency distribution trend. Figure 2b shows that pink noise had certain trends and regularities in its distribution, with a longer continuity. In the frequency domain, the energy distribution of pink noise decreased as the frequency increased. Therefore, the pink noise contained more information and had more complex dynamic and frequency variation characteristics.

**Figure 3:**
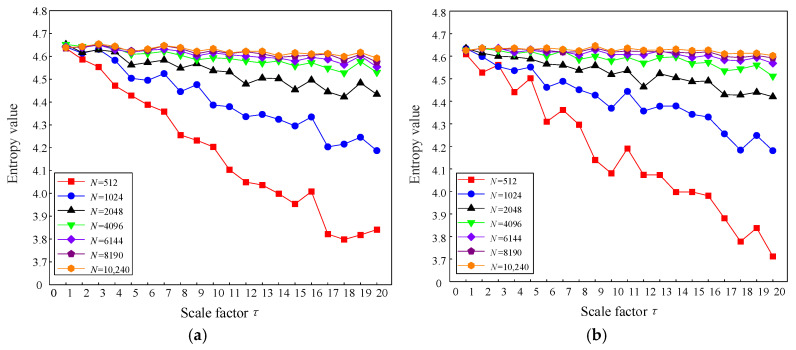
Comparison of TSMIE under different data scales. (**a**) White noise; (**b**) pink noise.

In order to analyze the influence of the data length on TSMIE for both types of signals, this paper calculated the TSMIE values of the two signals using seven different data lengths, with the remaining parameters set as m=2, R=4, and τ=20. Figure 3 displays the TSMIE value distribution results of the two noise signals at different scales.

From Figure 3, it can be observed that both white noise and pink noise exhibited significant fluctuations in TSMIE with respect to N=512,1024,2048. However, as the number of data points increased, TSMIE gradually stabilized. When N was greater than 4096, the influence of the data length on TSMIE decreased. Considering that larger data would increase the time cost, this paper set the data length to 4096.

**Figure 4:**
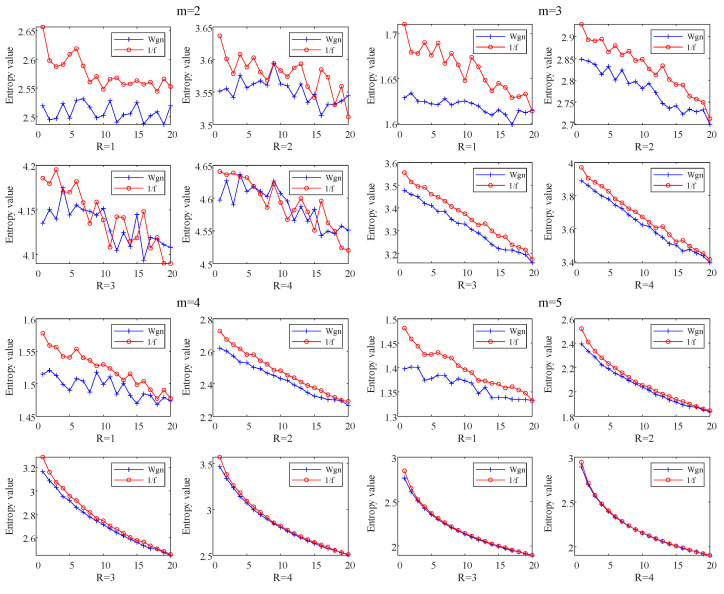
Comparison of TSMIE under different *m* and *R* values.

To analyze the influence of the parameters m and N on TSMIE, this paper calculated the TSMIE values of white noise and pink noise signals when m=2,3,4,5 and R=1,2,3,4. The results are shown in Figure 4.

Figure 4 shows that, with different parameters, the TSMIE values of both the white noise and pink noise signals gradually decreased as the scale factor increased. Due to the complex dynamic variation characteristics of pink noise, its TSMIE value was greater than that of white noise. This indicates that TSMIE can accurately assess the complexity of signals. Additionally, it can be noted that, under the same scale factor, the TSMIE differences between the two signals gradually decreased as *m* and *R* increased, making it difficult to distinguish between them. When m=3, the TSMIE values of the two signals were effectively distinguished; however, the TSMIE values fluctuated significantly when R=1,2. Considering both the distinctiveness and stability of TSMIE values, this paper set m=3 and R=3.

**Figure 5:**
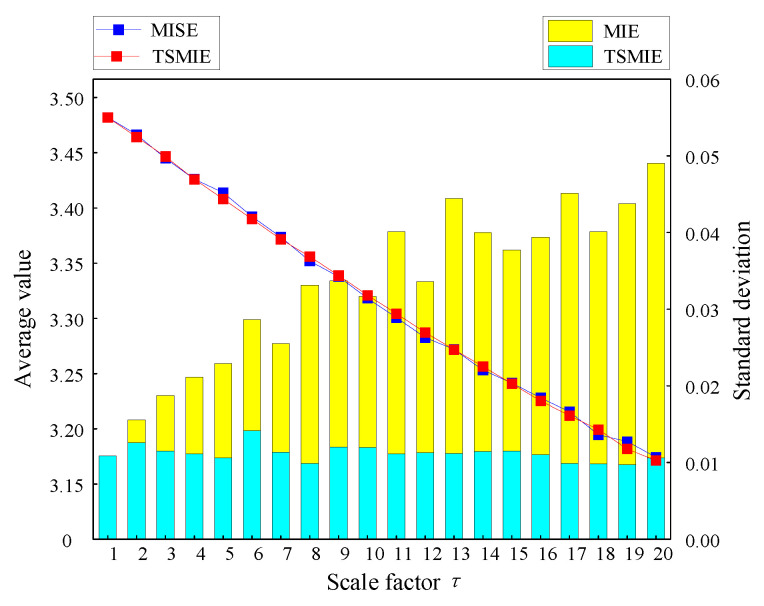
Comparison of means and standard deviations of MIE and TSMIE.

After setting the parameters, this paper calculated the multiscale increment entropy (MIE) and TSMIE of 100 sets of Gaussian white noise, and compared the average values and standard deviations. The results obtained are shown in Figure 5.

Figure 5 shows that, although the mean curves of MIE and TSMIE were similar, the standard deviation of TSMIE at all scales was smaller than that of MIE. This indicates that TSMIE produces a more stable estimation of signal dynamic changes and can extract representative signal features. The TSMIE index is more conducive to the feature extraction of signals, which can allow for the deep exploration of feature information.

### 2.3. CatBoost Model

CatBoost is an improved machine learning algorithm based on gradient boosting decision trees (GBDTs) that was developed by the Russian company Yandex in 2017 [29]. This algorithm employs symmetric decision trees as base learners, effectively addressing issues such as gradient bias, prediction shifts, and overfitting in traditional algorithms. CatBoost exhibits a high training efficiency and generalization capability. It is applicable to various classification and regression problems in different domains. It can handle different data types, providing accurate predictions and decision support, thereby providing broad application prospects. The principles of CatBoost are as follows [30].

During model training, the pre-processing of categorical features is required. In the gradient boosting algorithm, for low-dimensional categorical features, one-hot encoding is commonly used to convert discrete features into numerical features. However, when the dimensionality of the input categorical features is high, one-hot encoding may lead to the problem of dimension explosion. In such cases, the target statistic (TS) is employed to group categorical features. The TS estimates the expected value of each category’s target variable, which is used as a new numerical variable to replace the original categorical variable. In the GBDT algorithm, the average of the replaced category labels is used as the criterion for node splitting during the decision tree construction, known as the greedy target-based statistic (Greedy TS) algorithm.

However, Greedy TS has some limitations. When the data contain more information than labels, replacing categorical features with the average of all labels may lead to conditional shifts in the testing and training sets. Therefore, CatBoost is introduced to optimize Greedy TS by sorting all the data to construct multiple random sequences. During training, the labels’ averages replace the category of a specific feature sequence. Additionally, smoothing processing is applied by adding a prior distribution term to eliminate the impact of low-frequency categorical features and noise on the dataset. The optimized Greedy TS algorithm is defined in Equation (15).

(15) $${\tilde{x}}_{i,k}=\frac{\sum_{j=1}^{p-1}[x_{\sigma_{j},k}=x_{\sigma_{p},k}]\cdot Y_{j}+a\cdot p}{\Sigma_{j=1}^{p-1}[x_{\sigma_{j},k}=x_{\sigma_{p},k}]+a}$$

where ${\tilde{x}}_{i,k}$ is the k feature of the i sample in the original dataset. σ is the sequence of the original data that have been resorted. p is the prior term, which is the average of the labels in the dataset. $x_{\sigma_{j},k}$ is the k feature of the j sample after sorting. $x_{\sigma_{p},k}$ is the k feature of the prior term after sorting. $Y_{j}$ is the label value of the sample after sorting. a is the model weight coefficient, which is greater than 0.

Using the optimized Greedy TS for categorical feature encoding, CatBoost employs a greedy strategy to randomly combine any categorical features to generate new features. This is to better capture high-order data information. Besides generating the first split of the tree, CatBoost combines all the categorical features in the current tree with all the categorical features in the dataset, converting the combined categorical features into numerical features.

During the iteration of GBDT, the model constructs the base learners by calculating the gradient of the model with respect to the loss function. However, this method may result in gradient estimation errors and prediction shifts, leading to overfitting. Therefore, CatBoost employs ordered boosting to improve the model’s generalization capability and reduce the risk of overfitting. The calculation process of the ordered boosting is as follows.

Assuming that the number of trees is I, a training sample set ${\left(X_{k},Y_{k}\right)}_{k=1}^{n}$ contains n samples, where *X_k_* is the sample feature set and $Y_{k}$ is the sample label. The ordered boosting first initializes the model $M_{i}$, where i=1,2,3,…,n. Then, random sequences are generated and sorted. Next, each sample is iterated and each tree is constructed. The principle of model construction is to calculate the gradient of the first k−1 samples and train the model. For each feature set $X_{k}$, $M_{i}$ is corrected using $M_{i}=M_{i}+\Delta M$. Finally, the final model $M_{n}$ is obtained through the above loop iteration.

CatBoost employs a symmetric tree as the base predictor. During the iterative computation, the same splitting criterion is used for the same tree layer, making the left and right sub-trees completely symmetrical. Compared to traditional decision trees, this structure is more balanced and the computation speed is faster, effectively avoiding overfitting issues.

## 3. A Transformer Fault Diagnosis Model Based on TSMIE–CatBoost

By combining the effectiveness of TSMIE in feature extraction and the advantages of the CatBoost model in data classification, this paper proposes a TSMIE–CatBoost-based transformer vibration signal fault diagnosis model. The specific steps are as follows:

Step 1: Collect the vibration signals of the transformer under normal operation and different fault conditions. Normalize the data and construct the dataset.

Step 2: Perform a time-shift transformation on each time series in the dataset and calculate the information entropy (IE) of each shifted subsequence to obtain the TSMIE value of the transformer under normal and fault conditions.

Step 3: Set category labels based on the normal and fault conditions of the transformer and divide the feature vectors into training and testing sets. Input the training samples into the CatBoost model for training to obtain the TSMIE–CatBoost fault diagnosis model. Use the trained model for fault identification on the testing samples and output the identification results.

**Figure 6:**
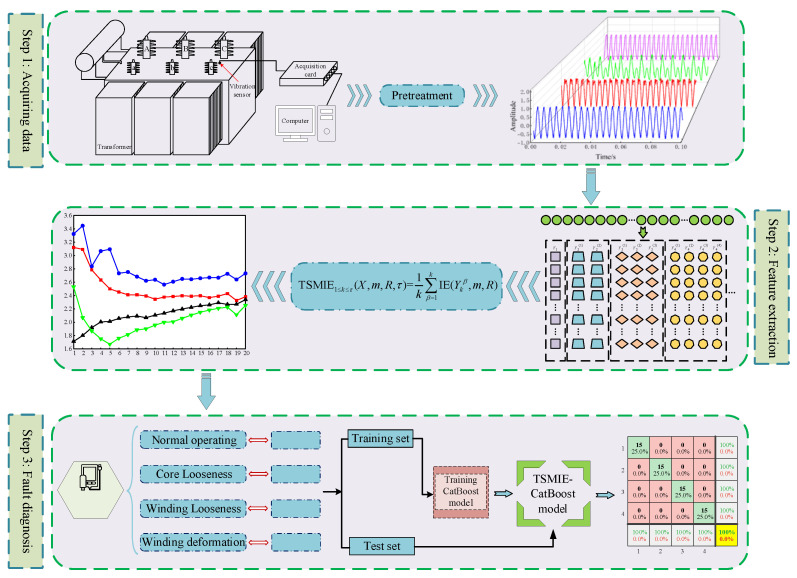
The transformer fault diagnosis procedure based on TSMIE–CatBoost.

The overall process is shown in Figure 6.

## 4. Signal Analysis

### 4.1. Transformer Vibration Signal Collection

**Figure 7:**
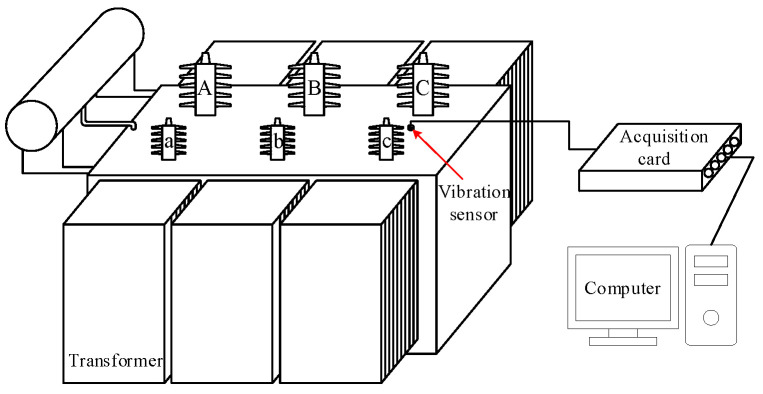
The signal acquisition device connection.

To validate the effectiveness of the proposed model on actual signals, the vibration signals of the transformer under four experimental conditions were collected, including normal operation (NO), core looseness (CL), winding looseness (WL), and winding deformation (WD). The parameters of the experimental transformer are shown in Table 2. And the connection of the signal acquisition device is shown in Figure 7.

The connection of the signal acquisition device is depicted in Figure 7. This setup includes the following key components:

Transformer (SZ-20,000/35): The experimental transformer under test, with specifications as described in Table 2.

The sensors used in this experiment were piezoelectric accelerometers, model ICP 601A01, with a sensitivity of 100 mv/g, a sampling frequency range of 0.27 Hz~10 kHz (±10%), a range of ±50 g, a working temperature of −54~121 °C, a weight of 80 g, and an output mode of top end. These sensors were placed at strategic points on the transformer to ensure accurate signal collection.

Data acquisition system: This system collected the signals from the accelerometers. The system was set to a sampling frequency of 10 kHz to capture high-resolution vibration data.

Computer Interface: The collected data were transferred to a computer for storage and further analysis. This interface ensured that the data could be processed and analyzed using the TSMIE–CatBoost model.

Figure 7 demonstrates how the sensors were connected to the transformer and the data acquisition system, and how the data flowed from the sensors to the computer for the analysis. This setup ensured that the vibration signals from the transformer under various conditions were accurately captured for the diagnosis model.

**Figure 8:**
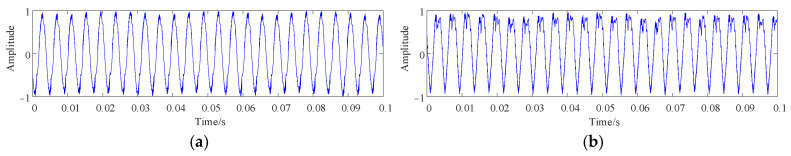

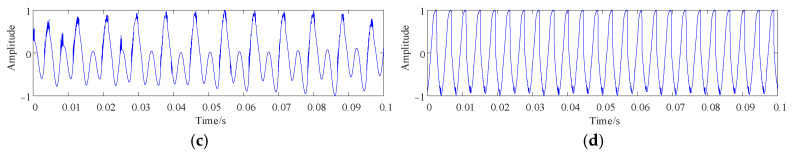
Transformer experimental vibration signals: (**a**) normal operation; (**b**) core looseness; (**c**) winding looseness; and (**d**) winding deformation.

The vibration signals of the transformers were extracted during light-load operation and normalized. The signals of the transformer under normal and fault conditions are shown in Figure 8.

Figure 8 shows that the vibration signals of the transformer under normal and fault conditions exhibited distinct characteristics. However, it was not possible to directly distinguish and evaluate the raw vibration signals effectively. The further extraction of the meaningful information contained in the transformer vibration signals is needed.

### 4.2. TSMIE Feature Extraction

**Figure 9:**
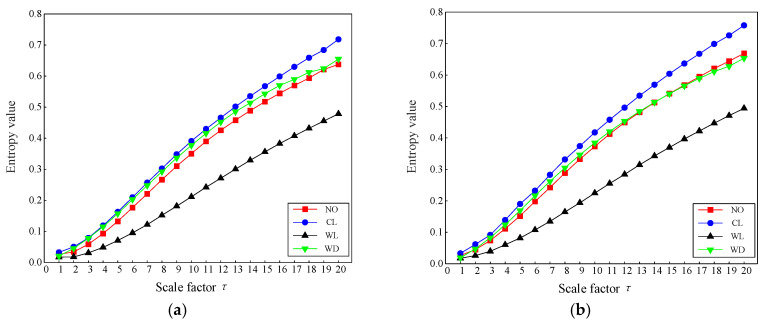

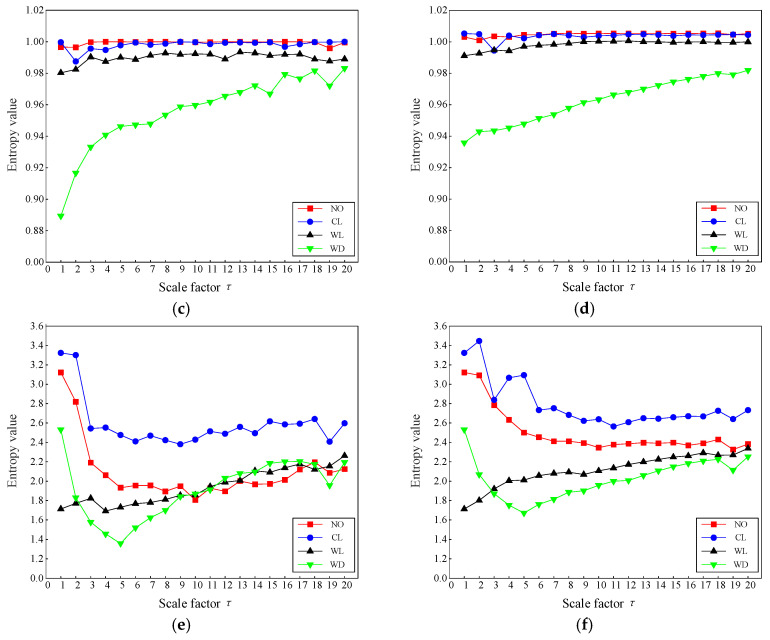
Multiscale entropy values of transformer vibration signals under normal and fault conditions: (**a**) MFE; (**b**) TSMFE; (**c**) MPE; (**d**) TSMPE; (**e**) MIE; and (**f**) TSMIE.

In this section, the collected transformer vibration signals underwent feature extraction. To highlight the superiority of TSMIE, multiscale fuzzy entropy (MFE), time-shift multiscale fuzzy entropy (TSMFE), multiscale permutation entropy (MPE), time-shift multiscale permutation entropy (TSMPE), and multiscale information entropy (MIE) were introduced for the comparative analysis. The parameter settings for the aforementioned entropy values are shown in Table 3, and the calculated entropy value distributions are presented in Figure 9.

Figure 9a shows that MFE clearly distinguished the winding looseness condition, while there was overlap among the normal operation, core looseness, and winding deformation conditions. As shown in Figure 9b, TSMFE and MFE had a similar entropy distribution, indicating that the time-shift operation did not significantly improve MFE. As shown in Figure 9c, MPE showed overlap among the first three conditions and only distinguished the winding deformation. The TSMPE distribution in Figure 9d is similar to that of MPE with less fluctuation, indicating that the time-shift operation can enhance the stability of feature extraction. As shown in Figure 9e, MIE effectively distinguished the normal and fault conditions at the initial scales, demonstrating that incremental entropy has an advantage in capturing useful information. However, the traditional coarse-graining method led to some information loss as the scale factor increased, resulting in overlap among three conditions at later scales. As shown in Figure 9f, TSMIE clearly distinguished the normal and fault conditions, enabling the extraction of more detailed and comprehensive vibration signal features. In summary, the comparison of multiscale entropy curves showed that the time-shift operation has significant advantages in reducing errors and enhancing the stability of feature extraction, validating the effectiveness of the proposed method.

**Figure 10:**
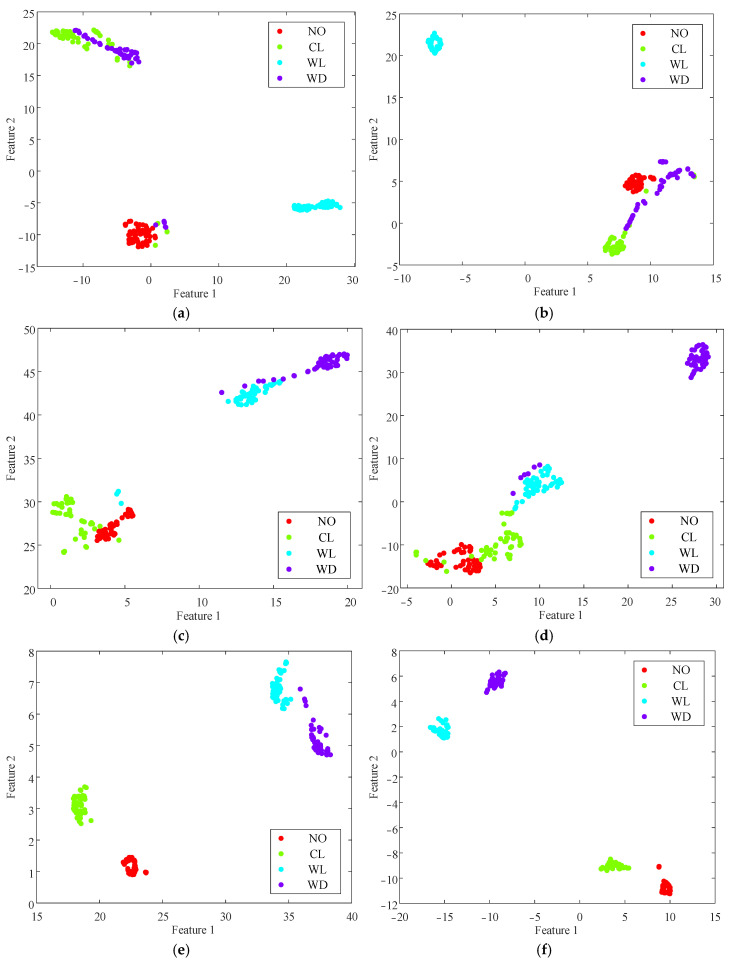
Multiscale entropy values after dimension reduction: (**a**) MFE; (**b**) TSMFE; (**c**) MPE; (**d**) TSMPE; (**e**) MIE; and (**f**) TSMIE.

Due to the high dimensionality of the multiscale entropy feature values mentioned above, direct processing could burden the classifier [31]. Therefore, to further validate the discriminative ability of the proposed features for the normal and fault conditions of a transformer, this study employed T-distributed stochastic neighbor embedding (T-SNE) for a dimensionality reduction analysis of the aforementioned entropy values. The results are presented in Figure 10.

It can be observed from Figure 10 that, after the dimension reduction using T-SNE, the differentiation effect of the different fault conditions aligned with the results in Figure 9. Specifically, MFE and TSMFE could only distinguish the winding looseness. MPE and TSMPE could distinguish the winding deformation, which still partially overlapped with the winding looseness. MIE could distinguish the normal operation from the core looseness, but there was still partial overlap between the winding looseness and the winding deformation. TSMIE showed good differentiation among the normal and fault conditions, and the aggregation of data for each condition was better than that of the previous five multiscale entropy values.

The operating environment of transformers is complex, and the vibration signals generated by the cooling system can mix with those on the surface of the transformer casing. The collected vibration signals contained certain noise, which necessitated that the extracted features exhibit noise resistance. Therefore, this study added Gaussian white noise with intensities of 15 dB, 20 dB, and 25 dB to the vibration signals under the normal and fault conditions. The MFE, TSMFE, MPE, TSMPE, MIE, and TSMIE values of each signal were calculated and compared. To investigate the efficiency of the proposed feature extraction method, this study compared the feature extraction times of different entropy values. The results are shown in Table 4.

It can be observed from Table 4 that, among different multiscale entropy values, MFE took the longest time due to the low efficiency during feature extraction. MIE had the shortest feature extraction time under different noise conditions. Since the time-shift operation required constructing new time-shifted subsequences, it increased the computational cost. However, among the time-shifted multiscale entropy measures, TSMIE took the shortest time. This indicates that incremental entropy has a higher efficiency in feature extraction compared to other entropy values.

### 4.3. Transformer Vibration Signal Fault Diagnosis

**Figure 11:**
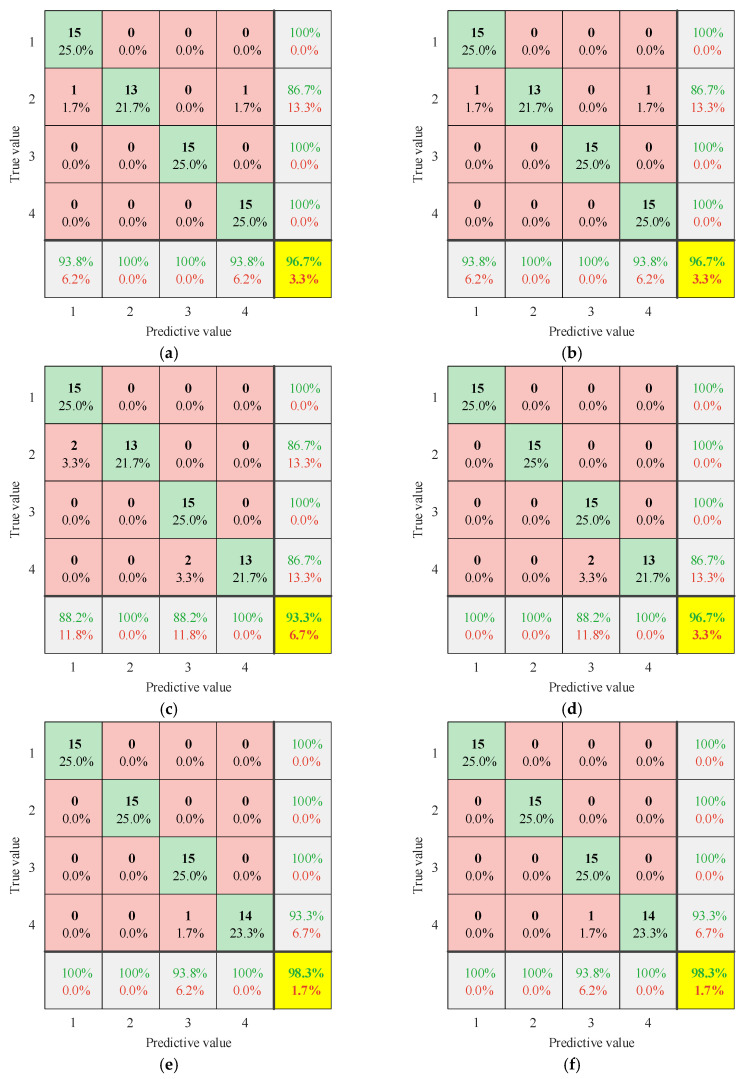
The confusion matrices of different multiscale entropy values: (**a**) MFE; (**b**) TSMFE; (**c**) MPE; (**d**) TSMPE; (**e**) MIE; and (**f**) TSMIE.

After obtaining the entropy features under the normal and fault conditions, this paper set the labels for normal operation, core looseness, winding looseness, and winding deformation as 1, 2, 3, and 4, respectively. A total of 70% of the dataset was used for training, while 30% was used for testing. The training samples were sent into CatBoost for model training, with the data labels used as the output for the fault diagnosis. The CatBoost parameters were set as follows: the iterations = 500, the learning rate = 0.01, the maximum tree depth = 6, and the L2 regularization parameter (L2_leaf_reg) = 3. The remaining parameters were set to the default values. To verify the advantage of the TSMIE–CatBoost model in transformer fault diagnoses, the other entropy values were also used for comparison. The confusion matrices for each model are shown in Figure 11.

In the confusion matrices shown in Figure 11, the values in green of the last column represent the recall rate for each condition, indicating the proportion of actual true samples that the model successfully predicted as true. The values in red represent the false positive rate for each condition, indicating the proportion of actual false samples that were incorrectly predicted as true. The values in green of the last row represent the precision rate for each condition, indicating the proportion of samples predicted as true that were actually true. The values in red represent the false negative rate for each condition, indicating the proportion of actual true samples that were incorrectly predicted as false. The values in green in the yellow box represent the model’s accuracy, one of the most intuitive evaluation metrics, indicating the proportion of correctly predicted samples out of the total samples. This was used to assess the model’s overall predictive ability. The values in red represent the model’s error rate, indicating the proportion of incorrectly predicted samples out of the total samples. Higher recall, precision, and accuracy rates, along with lower false positive, false negative, and error rates, indicated a better fault recognition capability and reliability of the model.

As shown in Figure 11, based on the superior performance of CatBoost, all the models had a recognition accuracy above 90%. The MIE–CatBoost model achieved a recognition accuracy of 98.3%, with only one winding deformation fault (label 4) being misidentified as winding looseness (label 3). The MFE–CatBoost and TSMFE–CatBoost models both achieved a recognition accuracy of 96.7%, with one core looseness fault (label 2) being misidentified as normal operation (label 1) and another as winding deformation (label 4). The MPE–CatBoost model had a fault recognition accuracy of only 93.3%, with two core looseness faults (label 2) misidentified as normal operation (label 1) and two winding deformation faults (label 4) misidentified as winding looseness (label 3). However, the fault recognition accuracy of the TSMPE–CatBoost model improved through entropy enhancement, rising to 96.7%, with only two winding deformation faults (label 4) being misidentified. In the identification of the four conditions, the TSMIE–CatBoost model achieved a recall and precision rate of 100%, with false positive and false negative rates of 0%. These results indicate that the TSMIE–CatBoost model demonstrates a high diagnostic accuracy and stability for transformer vibration signals, accurately identifying normal and fault conditions of the transformer. Comparing the diagnostic rates of different entropy models revealed that TSMIE not only compensated for the inability of MFE and MPE, but also overcame the issue of information loss in the signal amplitude caused by the insufficient granularity of MIE.

To verify the robustness of the proposed method against noise, the transformer vibration signals with added noise from Section 4.1 were input into each model. The diagnostic accuracy of the different models is shown in Table 5.

From Table 5, it can be concluded that the TSMIE–CatBoost model achieved the highest diagnostic accuracy across the datasets with different noise levels. Even under strong noise (SNR = 15 dB), its fault diagnosis rate reached 96.67%, which was 8.34% higher than that of traditional entropy values. This indicates that the TSMIE–CatBoost model not only had a high feature extraction efficiency, but it also achieved a good diagnostic performance in noisy environments.

### 4.4. Comparative Analysis of Different Fault Diagnosis Models

To further verify the superior performance of the TSMIE–CatBoost model in transformer fault diagnoses, this paper selected TSMFE, TSMPE, and TSMIE under the normal and fault conditions of the transformer and sent them into a back propagation neural network (BPNN), support vector machine (SVM), random forest (RF), and K-nearest neighbor (KNN) for fault diagnoses. The parameters are shown in Table 6.

**Figure 12:**
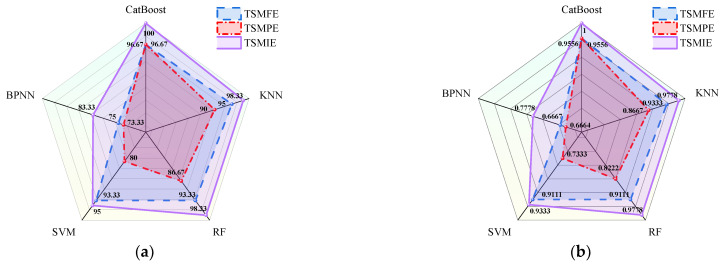
Distribution of recognition accuracy and Kappa coefficients of different models: (**a**) recognition accuracy; (**b**) *Kappa* coefficients.

After obtaining the diagnostic results of different models, this paper introduced the accuracy and *Kappa* coefficient, as well as the recall, precision, false negative rate, and false positive rate from the confusion matrix, to evaluate the model performance. The *Kappa* coefficient is a metric that measures the consistency between the predicted values and the actual observed values. It is used to assess the consistency and robustness of the classifier, whose value ranges from −1 to 1, with values closer to 1 indicating a better diagnostic performance. The calculation of the *Kappa* coefficient is defined in Equations (16) and (17).

(16) $$Kappa=\frac{Accuracy-P_{e}}{1-P_{e}}$$

(17) $$Accuracy=\frac{TP+TN}{TP+TN+FP+FN}$$

(18) $$P_{e}=\frac{\sum_{i=1}^{n}a_{i}\times b_{i}}{n\times n}$$

where Accuracy represents the accuracy of the model’s recognition. TP represents true positive, TN represents true negative, FP represents false positive, and FN represents false negative. $a_{i}$ is the number of actual samples for each class. $b_{i}$ is the number of predicted samples for each class. n is the total number of samples. The accuracy and *Kappa* coefficient of different models are shown in Figure 12. The recall, precision, false negative rate, and false positive rate for different conditions in the confusion matrices of each model were calculated and averaged, as shown in Table 7.

From Figure 12, it can be observed that, across different classifiers, CatBoost achieved the highest accuracy and Kappa coefficients. This indicates that the CatBoost model possesses more precise recognition capabilities and stronger generalization abilities for transformer vibration fault diagnoses. TSMIE consistently achieved the highest accuracy and Kappa coefficients across all the classifiers. This suggests that TSMIE accurately and comprehensively describes the vibration signals of transformers under normal and fault conditions, demonstrating excellent feature extraction capabilities.

Table 7 indicates that TSMIE consistently exhibited the highest recall and precision and the lowest false negative and false positive rates across different models. Among the classifiers, CatBoost demonstrated the best performance, especially with the TSMIE–CatBoost model, which achieved a recall and precision rate of 100% and a false negative and false positive rate of 0%. This means that the recognition capability of the proposed model was more stable and accurate compared to the other models.

## 5. Conclusions

This paper proposes a fault diagnosis model combining time-shift multiscale increment entropy (TSMIE) with CatBoost, achieving transformer fault diagnoses under different operating conditions. The conclusions are as follows.

1. By addressing the issue of signal loss and incomplete feature extraction in the coarse-graining process of MIE, this paper introduces TSMIE by integrating the concept of time-shift. The influence of different parameters on TSMIE was analyzed by applying simulation signals, and its optimal parameters were determined. Finally, a comparison was made with MIE in terms of the means and standard deviations. The results showed that TSMIE had a much smaller standard deviation than MIE, which could provide a more stable description of signal dynamic changes, verifying TSMIE’s good signal feature extraction ability and stability.
2. By utilizing TSMIE for feature extraction from vibration signals under normal and fault conditions of a transformer, comparative analyses were conducted with MFE, TSMFE, MPE, TSMPE, and MIE in terms of the entropy distribution, dimension reduction visualization, and noise robustness. The results showed that, compared with other entropy values, TSMIE can effectively distinguish normal operation, iron core looseness, winding looseness, and winding deformation faults in a transformer. After a T-SNE dimensionality reduction, the data aggregation degree of TSMIE was the best, and it could effectively distinguish the four situations. Moreover, TSMIE had the shortest running time and the highest diagnostic accuracy under different noise conditions, verifying the accuracy and noise robustness of TSMIE in extracting features from transformer vibration signals.
3. CatBoost was integrated with TSMIE for the transformer vibration fault diagnosis. Comparative analyses were conducted with the MFE–CatBoost, TSMFE–CatBoost, MPE–CatBoost, TSMPE–CatBoost, and MIE–CatBoost models. BPNN, SVM, RF, KNN, and CatBoost were also compared for transformer fault diagnoses. The results showed that the TSMIE–CatBoost model exhibited a good performance in its accuracy and Kappa coefficient, as well as its recall rate, precision rate, false negative rate, and false positive rate in the confusion matrix, verifying the superiority of the proposed model for transformer fault diagnoses. This indicates that the fault diagnosis model proposed in this paper is an excellent method and provides new ideas for transformer fault diagnoses.

The research in this paper was conducted on a single transformer. However, the operating conditions of transformers are complex, and there are differences in the structure of different transformers. Moreover, different signal acquisition points have an impact on vibration signals. At the same time, insufficient transformer fault sample data can also affect the generalization ability and robustness of the model. Therefore, future research will focus on diverse transformer vibration signal collection from different operating conditions, transformers, and measurement points to expand the dataset and improve the effectiveness and universality of the proposed method.

## Figures and Tables

**Table 1:** Advantages and disadvantages of traditional machine learning methods.

Method	Advantages	Disadvantages
BPNN	Strong non-linear mapping capability; capable of handling non-linear and complex problems; excellent adaptability and versatility	Long training time; slow convergence speed; prone to local optima; sensitive to parameters; requires manual setting of network structure
SVM	Strong generalization ability; suitable for small-size, non-linear, and high-dimensional data	Long learning time; sensitive to parameters; requires data preprocessing
ELM	Fewer parameters; fast training speed; strong generalization ability; robust to noise	Weak stability; limited ability to process complex nonlinear data; unsuitable for data with special distributions
KNN	Requires no repeated model training; robust to noise	Sensitive to data dimensions; poor performance in handling high-dimensional and imbalanced data; slow convergence speed

**Table 2:** Transformer parameters.

Parameter	Values
Model	SZ-20,000/35
Type	Three-phase
Rated Power	20,000 kVA
Rated Voltage	35 ± 3 × 2.5%/10 kV
No-load current	0.43%
Impedance voltage	8%

**Table 3:** Parameter settings for different types of multiscale entropy.

Category	Embedding Dimension *m*	Similarity Tolerance *r*	Gradient Parameter *n*	Time Delay d	Resolution Parameter *R*
MFE	2	0.15 SD	2	1	—
TSMFE	2	0.15 SD	2	1	—
MPE	2	—	—	1	—
TSMPE	2	—	—	1	—
MIE	3	—	—	—	3
TSMIE	3	—	—	—	3

**Table 4:** The extraction times for different types of entropy (Unit: s).

	MFE	MPE	MIE	TSMFE	TSMPE	TSMIE
Noiseless	274.89	10.92	8.56	824.78	93.70	85.17
SNR = 15 dB	278.95	9.73	8.93	824.45	100.56	94.78
SNR = 20 dB	291.12	10.07	8.98	883.72	89.58	88.39
SNR = 25 dB	292.78	10.25	8.73	883.63	84.18	85.78

**Table 5:** The diagnostic accuracy of different models.

Diagnostic Model	SNR = 15	SNR = 20	SNR = 25
MFE–CatBoost	88.33%	93.33%	93.33%
TSMFE–CatBoost	88.33%	96.67%	96.67%
MPE–CatBoost	86.67%	86.67%	91.67%
TSMPE–CatBoost	93.33%	96.67%	96.67%
MIE–CatBoost	96.67%	96.67%	96.67%
TSMIE–CatBoost	96.67%	98.33%	98.33%

**Table 6:** The parameters of different methods.

Parameters	BPNN	SVM	RF	KNN
Network structure	20-2-4	-	-	-
Iterations	1000	-	-	-
Learning rate	0.01	-	-	-
Target training error	0.000001	-	-	-
Kernel function	-	RBF	-	-
Kernel function parameter	-	0.01	-	-
Penalty factor	-	10	-	-
Number of decision trees	-	-	10	-
Minimum number of leaves	-	-	5	-
Number of nearest neighbor samples	-	-	-	4

**Table 7:** The recall rate, precision rate, false negative rate, and false positive rate of different models.

Diagnostic Model	Recall Rate	Precision Rate	False Negative Rate	False Positive Rate
TSMFE–BPNN	75%	60.45%	25%	14.55%
TSMPE–BPNN	73.33%	60.51%	26.67%	14.49%
TSMIE–BPNN	83.33%	90%	16.67%	10%
TSMFE–SVM	93.33%	94.12%	6.67%	5.88%
TSMPE–SVM	80%	83.77%	20%	16.23%
TSMIE–SVM	95%	95.83%	5%	4.17%
TSMFE–RF	93.33%	94.27%	6.67%	5.73%
TSMPE–RF	86.67%	89.58%	13.33%	10.42%
TSMIE–RF	98.33%	98.44%	1.67%	1.56%
TSMFE–KNN	95%	95.5%	5%	4.5%
TSMPE–KNN	90%	91.8%	10%	8.21%
TSMIE–KNN	98.33%	98.44%	1.67%	1.56%
TSMFE–CatBoost	96.67%	96.88%	3.33%	3.13%
TSMPE–CatBoost	96.67%	97.06%	3.33%	2.94%
TSMIE–CatBoost	100%	100%	0%	0%

## Data Availability

The data that support the findings of this study are not publicly available due to the confidentiality requirements of one ongoing project.
